# Assessment of Corneal Epithelial Thickness in Asymmetric Keratoconic Eyes and Normal Eyes Using Fourier Domain Optical Coherence Tomography

**DOI:** 10.1155/2016/5697343

**Published:** 2016-06-09

**Authors:** S. Catalan, L. Cadarso, F. Esteves, J. Salgado-Borges, M. Lopez, C. Cadarso

**Affiliations:** ^1^Department of Ophthalmology, University Hospital of Vigo, 36200 Vigo, Spain; ^2^Clínica Cadarso, 36203 Vigo, Spain; ^3^Department of Ophthalmology, Hospital da Boa Nova, 4455-421 Matosinhos, Portugal; ^4^Clínica Salgado-Borges, 4000-422 Porto, Portugal; ^5^Department of Statistics and Operations Research, School of Medicine, University of Santiago de Compostela, 15782 Santiago de Compostela, Spain

## Abstract

*Purpose*. To compare the characteristics of asymmetric keratoconic eyes and normal eyes by Fourier domain optical coherence tomography (OCT) corneal mapping.* Methods*. Retrospective corneal and epithelial thickness OCT data for 74 patients were compared in three groups of eyes: keratoconic (*n* = 22) and normal fellow eyes (*n* = 22) in patients with asymmetric keratoconus and normal eyes (*n* = 104) in healthy subjects. Areas under the curve (AUC) of receiver operator characteristic (ROC) curves for each variable were compared across groups to indicate their discrimination capacity.* Results*. Three variables were found to differ significantly between fellow eyes and normal eyes (all *p* < 0.05): minimum corneal thickness, thinnest corneal point, and central corneal thickness. These variables combined showed a high discrimination power to differentiate fellow eyes from normal eyes indicated by an AUC of 0.840 (95% CI: 0.762–0.918).* Conclusions*. Our findings indicate that topographically normal fellow eyes in patients with very asymmetric keratoconus differ from the eyes of healthy individuals in terms of their corneal epithelial and pachymetry maps. This type of information could be useful for an early diagnosis of keratoconus in topographically normal eyes.

## 1. Introduction

Keratoconus is a bilateral, noninflammatory corneal ectasia in which the cornea assumes a conical shape due to progressive thinning and steepening of the corneal stroma. With a prevalence of 54 per 100 000, it is the most common primary corneal ectasia [1].

Moderate forms of keratoconus are easy to detect using several devices [2] to examine anterior corneal topography. These range from simple inexpensive devices, such as handheld keratoscopes (Placido's disks), to sophisticated devices such as computer-assisted videokeratoscopes. In clinical practice, the Pentacam corneal tomographer [3] or Orbscan topography system [4] is widely used to detect subtle changes and control disease progression.

In contrast, the diagnosis of subclinical keratoconus is a challenge and this can have dire consequences. For example, an undetected incipient ectasia could be worsened by a refractive procedure such as LASIK, whereby the already reduced mechanical strength of the cornea is further weakened by surgery possibly causing rapid progression of the ectasia [5].

Recently, it has been noted that the cornea of keratoconic eyes may show early thickness changes involving both the stroma and epithelium. Such observations include progressive thinning of the stroma and a localized area of thinner epithelium over the cone surrounded by an annulus of thicker epithelium [6]. Several methods have been used to map the corneal epithelium [6–8].

The present study sought to detect subtle changes in early stages of keratoconus. Corneal epithelial thickness and total corneal thickness measurements were made in healthy eyes and in the eyes of patients with highly asymmetric keratoconus. We propose that in these patients the topographically normal eye is a good model to assess early changes, since the vast majority of these fellow eyes will develop keratoconus [9]. Although other authors have also used this model to detect subclinical changes in keratoconus, these studies have examined other corneal properties [10, 11].

## 2. Materials and Methods

### 2.1. Subjects

Participants were recruited among the adult patients (>18 years) of two European healthcare centers: Clínica Cadarso (Vigo, Spain) and the Hospital of Santa Maria da Feira (Portugal). The study protocol adhered to the principles of the Declaration of Helsinki and received institutional review board approval.

All adult patients for which complete OCT corneal thickness and corneal epithelial thickness data were available were identified in the databases of the two participating centers.

Normal subjects were recruited from patients seeking refractive surgery and cataract surgery consultation.

Of the 160 eyes of 80 patients identified, two eyes with corneal scarring, seven eyes subjected to ocular surgery, and two eyes giving rise to segmentation errors (the OCT is not able to detect the corneal layers and their boundaries) were excluded. This left us with a final study sample of 148 eyes of 74 patients.

Corneal topography was obtained using a Placido-topography and an elevation-based Scheimpflug imaging device (Pentacam, Oculus).

The 148 eyes were divided into three groups according to slit-lamp findings and the topographical criteria for keratoconus (Placido disc-based indices) [12] described below:

(1) Normal eyes (*n* = 104): eyes showing normal slit-lamp findings and no topographical signs of keratoconus.
(2) Keratoconic eyes (*n* = 22): eyes of patients with diagnosed asymmetric keratoconus showing clinical and topographical findings compatible with keratoconus.
(3) Fellow eyes (*n* = 22): contralateral eyes of the patients with asymmetric keratoconus showing normal slit-lamp findings and no signs of topographic keratoconus.

### 2.2. Topographical Criteria for Keratoconus

1. Inferior-superior power asymmetry: difference between the average surface power of 5 inferior points and 5 superior points, 3 mm from the center of the cornea at 30° intervals (≥1.4).
2. Central corneal power: ≥47.2 diopters.
3. KISA% index: product of four indices in the topography (≥100%).
4. Keratoconus predictability index: linear discriminant analysis of 8 quantitative topographic indices (≥0.23).

### 2.3. Fourier Domain OCT and Image Analysis

The instrument used was Fourier domain OCT system (RTVue; Optovue, Inc., Fremont, CA) which was fitted with a corneal adaptor module for the corneal epithelial maps. This is a 26000 Hz Fourier domain OCT system with 5 microns of axial resolution. The corneal mapping scan pattern includes 6 mm lines on 8 meridians centered at the pupil and each line scans 1024 axial points in 0.04 seconds. The set of eight meridians is acquired in 0.31 seconds and the exam is repeated five times in 1.55 seconds. The software then generates the epithelium boundaries and the thickness map.

Each eye was scanned 3 times within a single visit. Maps reproducibility was assessed analyzing 3 acquisitions for each eye of each patient without finding major changes.

A computer algorithm automatically maps corneal thickness (across the central 5 mm of the corneal surface divided into three zones: central 2 mm, superior 2 to 5 mm, and inferior 2 to 5 mm) and calculates the following variables on the pachymetry and epithelial maps.

### 2.4. Pachymetry Map

 Superonasal − inferotemporal (SN − IT): difference between superonasal and inferotemporal corneal thickness (2 to 5 mm from the center).
 Minimum pachymetry (Min): thickness of the thinnest corneal point.
 Minimum − median (Min − Med): difference between the thickness of the thinnest point and the median of all points.
 Superior − inferior (S − I): difference between mean superior and mean inferior corneal thickness (2 to 5 mm from the center).
 
*Y* location: location of the thinnest point in the vertical meridian (positive values for locations superior to the corneal vertex; negative values for locations inferior to the corneal vertex).
 Minimum − maximum (Min − Max): difference between the thickest and thinnest point.
 Central pachymetry (CCT): corneal thickness at the central point.

### 2.5. Epithelial Map

 Superior epithelium (Sup): mean of thickness values recorded in the superior epithelium (2 to 5 mm from the center).
 Inferior epithelium (Inf_ep): mean of thickness values recorded in the inferior epithelium (2 to 5 mm from the center).
 Minimum epithelium (Min_ep): thinnest point of the epithelium.
 Maximum epithelium (Max): thickest point of the epithelium.
 Minimum − maximum (Min − Max_ep): difference between minimum and maximum epithelial thickness.
 Standard deviation (SD): standard deviation of all epithelial thicknesses recorded in the central 5 mm of the cornea.
 Central epithelial thickness (CET): thickness of the epithelium at the central point.

### 2.6. Statistical Analysis

Statistical analysis was performed on both qualitative variables (provided as frequencies and percentages) and quantitative variables (provided as the mean ± standard deviation and ranges). The Kolmogorov-Smirnov test was used to check the normality of the data. Differences in the diagnostic variables among the three groups of eyes were determined in pairwise comparisons conducted using the Kruskal-Wallis test and post hoc analysis (normal versus keratoconic eyes, normal versus fellow eyes, and keratoconic versus fellow eyes). An association was considered significant when *p* < 0.05.

The receiver operating characteristic (ROC) curve method was used to assess the diagnostic accuracy of each variable [13–16]. As a measure of the capacity of each variable to discriminate between normal and keratoconic eyes, the area under the ROC curve (AUC) was computed using cluster data, considering the eye (left or right) as a cluster (taking into account the possible correlation between both eyes of the same patient). When the relationship between the diagnostic variable and the presence of keratoconus was not monotonic (increasing or decreasing), a new transformed diagnostic variable was obtained by estimating the probability that the patient has keratoconus by means of Generalized Additive Models (GAMs) [17, 18] for binary data. A GAM is a flexible regression model used to express the nonlinear (smooth) effect of a continuous covariate on the response. In our case, the response is a binary variable that indicates whether the patient has keratoconus (=1) or not (=0), and the continuous covariate is the corresponding diagnostic variable for keratoconus. To assess improvements in discrimination capacity, a combination of several variables was also considered using Generalized Linear Models (GLMs) [19]. A GLM is a particular case of a GAM, in which the effect of the covariate on the presence of keratoconus is linear. All statistical tests were conducted with R 3.0.1 (R Development Core Team, 2013 [20]). ROC curves were constructed with the R package ROCR [21]. Logistic GAMs were fitted with the gam function of the R package mgcv [22]. To compute AUC with cluster data, considering the eye (left/right) as a cluster, a specific function in R was used (packages freely available at https://www.R-project.org/).

## 3. Results

Demographic data were mean age 37.96 ± 11.32 (18 to 61 years), 34 women (65.38%) and 18 men (34.62%) for the 52 subjects without keratoconus (104 eyes), and mean age 34.91 ± 11.96 (18 to 63 years), 6 women (27.27%) and 16 men (72.73%) for the patients with asymmetric keratoconus (*n* = 22, 44 eyes).

The OCT mapping data obtained for the three groups of eyes (keratoconic, fellow, and normal) are provided in Table 1.

**Figure 1:**
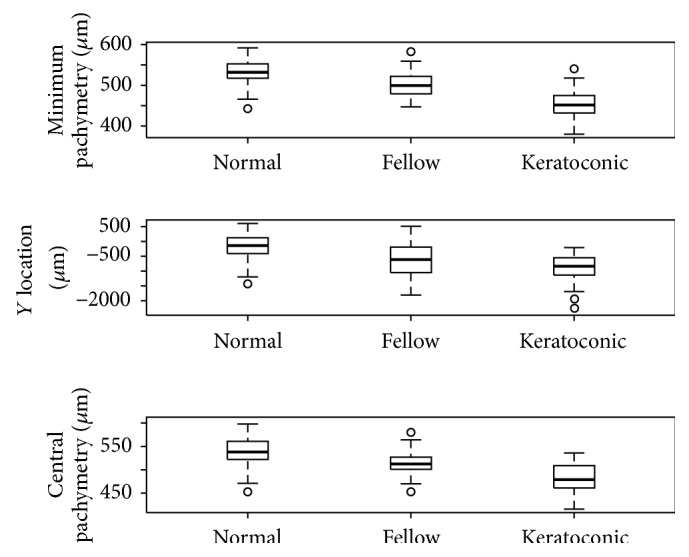
Values recorded for the variables Min,* Y* location, and CCT in the three study groups.

Pairwise comparisons among the three groups of eyes revealed the following significant differences: keratoconic versus normal eyes, all variables (*p* < 0.01); keratoconic versus fellow eyes, all variables except Sup (*p* = 0.101),* Y* location (*p* = 0.067), and Inf_ep (*p* = 0.05); and normal versus fellow eyes, Min,* Y* location, and central pachymetry (*p* < 0.001) (Figure 1).

AUC for all variables were 0.742 to 0.964 for keratoconic versus normal eyes indicating their good discrimination capacity (CI > 0.5) and 0.690 to 0.935 for keratoconic versus fellow eyes also indicating their good discrimination capacity (CI > 0.5). In the comparisons of fellow eyes versus normal eyes, the three variables showing the greater AUC were Min (AUC: 0.780; CI: 0.698–0.862),* Y* location (AUC: 0.725; CI: 0.474–0.976), and CCT (AUC: 0.765; CI: 0.713–0.816). The variables Min and CCT were able to discriminate well between the two sets of eyes whereas* Y* location showed a poor discrimination power (CI < 0.5).

**Figure 2:**
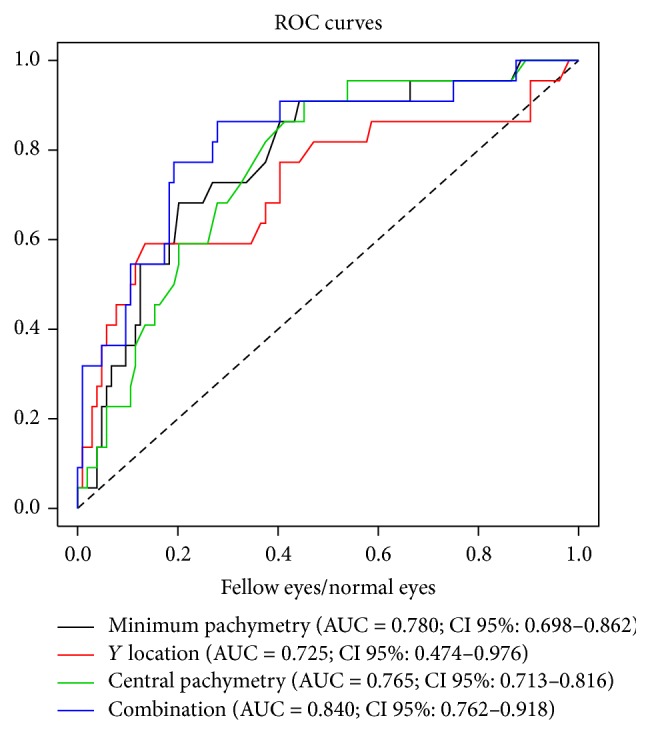
ROC curves and AUC for the three variables showing significant differences between normal and fellow eyes and for the model combining these three variables.

Finally, combinations of selected variables showing good discrimination capacity were tested, avoiding the introduction of correlated data in the same model. The following AUC were obtained for the different combinations of variables included in the models: keratoconic versus normal eyes, AUC = 0.974 (95% IC: 0.909–1.038) for Min − Med, superior − inferior, and Min_ep; keratoconic versus fellow eyes, AUC = 0.938 (95% CI: 0.928–0.948) for SD and CCT; and normal versus fellow eyes, AUC = 0.840 (95% CI: 0.762–0.918) for all variables. This last model including all variables emerged as showing the best discrimination power when compared with the AUC of each of the three variables found to vary significantly between normal eyes and fellow eyes (Min,* Y* location, and central pachymetry) (Figure 2).

## 4. Discussion

Our study was designed to identify possible subtle changes in the corneal epithelial thickness maps of patients with very early stage keratoconus. To assess this incipient stage of disease, we compared several corneal and epithelial variables in normal eyes and both the keratoconic eyes of patients with asymmetric keratoconus and their fellow eyes with no topographical signs of keratoconus.

Epithelial thickness profiles may increase the sensitivity and specificity of screening for keratoconus compared to corneal topography alone and may be useful in clinical practice. Epithelial information may allow for an earlier diagnosis of keratoconus, as epithelial changes will precede any changes produced on the front surface of the cornea [23]. Such epithelial thickness changes in keratoconus have been examined by other authors [6, 7]. Today, corneal epithelial and pachymetry profiles can be assessed through OCT, an accurate, rapid noninvasive tool [8].

In this study, we compared several epithelial and corneal variables extracted from Fourier domain OCT maps in the three groups of eyes described above. Significant differences were observed in all these variables between normal and keratoconic eyes, while when keratoconic and fellow eyes were compared, significant differences emerged for all variables except three (*Y* location, Sup, and Inf_ep). In contrast, when fellow and normal eyes were compared, differences were only observed in Min (*p* < 0.001; normal eyes 531.7 ± 30.17 *μ*m, fellow eyes 503.2 ± 32.71 *μ*m),* Y* location (*p* < 0.001; normal eyes −155.9 ± 398.43 *μ*m, fellow eyes −558.6 ± 591.70 *μ*m), and CCT (*p* < 0.001; normal eyes: 537.6 ± 30.66, fellow eyes 513.1 ± 31.90 *μ*m). The values obtained for each variable and differences observed between keratoconic and normal eyes are similar to those reported by Li et al. [8].

In addition, we examined the capacity of the variables determined to discriminate between the different groups of eyes. The variables showing best discrimination capacity were those that differed between keratoconic and normal eyes, followed by those observed to differ in the keratoconic eyes versus fellow eyes and then by those in the normal eyes versus fellow eyes. For this last comparison, we also detected a high differentiation capacity of a combination of variables indicated by an AUC of 0.840.

In a similar study, Temstet et al. compared OCT epithelial maps in keratoconic eyes, normal eyes, and eyes with forme fruste keratoconus showing no topographical or clinical signs of keratoconus. Significant differences between groups were detected for epithelial thickness in the thinnest corneal zone, the location of minimal epithelial thickness and minimum corneal thickness [24]. Although their sample size of keratoconic and fellow eyes was larger, the results reported by these authors are similar to those obtained in our study.

Reinstein et al. [25] assessed the effectiveness of an algorithm derived from Artemis very high frequency digital ultrasound to detect keratoconus by examining topographically normal fellow eyes in a series of patients with asymmetric keratoconus. The epithelial maps obtained by these authors were indicative of keratoconus in half the normal fellow eyes.

OCT measurements in our study include the thickness of the precorneal tear film. Tear film thickness values are comprised between 3 and 5 *μ*m [26–28].

Francoz et al. measure the precorneal tear film with spectral-domain OCT and they exclude it from the epithelial measurements, obtaining absolute lower values in comparison with other studies [29].

On the other hand, Reinstein et al. analyze epithelial thickness in the normal cornea with an Artemis VHF digital ultrasound. It was carried out using an ultrasonic standoff medium and so provides the advantages of immersion scanning and the precorneal tear film is not incorporated in the corneal or epithelial thickness measurements, obtaining more accurate results [30].

OCT epithelial and corneal measurements including the tear film thickness can lead to potential inaccuracies of the absolute values and this could be a limitation to our study.

## 5. Conclusions

In summary, along with complementary tests, corneal/epithelial thickness mapping by OCT could be useful to detect an incipient corneal ectasia in clinically and topographically normal eyes. This finding could have important implications for avoiding keratectasia when refractive surgery is performed on an apparently normal eye. Our findings provide direction for future studies designed to improve the early diagnosis of this disease by comparing other variables in larger series of patients with asymmetric keratoconus.

## Figures and Tables

**Table 1:** Data obtained for the three groups of eyes.

Normal eyes				
Variable	Mean	SD	Median	Range
SN − IT	17.39	12.92	17	−24 to 54
Min	531.7	30.17	532	454 to 592
Min − Med	−20.50	5.06	−20	−47 to −8
S − I	8.519	12.70	9	−40 to 48
*Y* location	−155.9	398.43	−141	−137 to 607
Min − Max	−53.42	11.81	−52	−97 to −26
CCT	537.6	30.66	538	459 to 598
Sup	52.03	3.27	52	37 to 60
Inf_ep	53.38	3.10	53	46 to 62
Min_ep	49.52	3.73	50	33 to 57
Max	56	3.55	56	48 to 69
Min − Max_ep	−6.423	3.04	−6	−25 to −2
SD	1.549	0.75	1.40	0.6 to 4.9
CET	52.90	3.09	53	42 to 61

Fellow eyes				
Variable	Mean	SD	Median	Range

SN − IT	24.50	20.05	20	−1 to 68
Min	503.2	32.71	499.5	447 to 597
Min − Med	−22.36	9.42	−20.5	−48 to −13
S − I	15.950	17.35	15	−19 to 48
*Y* location	−558.6	591.70	−610	−1809 to 513
Min − Max	−57.50	18.80	−52.5	−103 to −37
CCT	513.1	31.90	512.5	453 to 605
Sup	53.27	4.25	52	48 to 67
Inf_ep	53.14	4.50	52.5	47 to 70
Min_ep	49.09	4.74	49	37 to 64
Max	56.86	4.52	56.5	52 to 73
Min − Max_ep	−7.818	3.89	−6	−20 to −3
SD	1.891	0.99	1.50	0.7 to 4.7
CET	52	4.07	53.23	49 to 68

Keratoconic eyes				
Variable	Mean	SD	Median	Range

SN − IT	56.95	28.49	55	12 to 114
Min	458.0	45.14	451.5	380 to 589
Min − Med	−54.27	23.72	−51	−101 to −14
S − I	44.770	35.25	41	−38 to 115
*Y* location	−947.5	549.53	−834	−2369 to −206
Min − Max	−114.60	51.96	−99	−227 to −44
CCT	482.9	30.62	479	416 to 536
Sup	55.55	6.36	55	40 to 67
Inf_ep	50.82	5.86	49.5	41 to 64
Min_ep	41.18	6.45	42	33 to 56
Max	63.36	8.45	62	47 to 80
Min − Max_ep	−22.090	10.80	−20	−44 to −7
SD	5.918	3.19	5.55	1.7 to 13.8
CET	48.59	5.71	47.5	39 to 61
